# Associations of Prenatal Exposure to Triclosan and Maternal Thyroid Hormone Levels: A Systematic Review and Meta-Analysis

**DOI:** 10.3389/fendo.2020.607055

**Published:** 2021-01-13

**Authors:** Danrong Chen, Jiani Liu, Wu Yan, Kacey Fang, Yankai Xia, Wei Lv, Zhonghua Shi

**Affiliations:** ^1^ State Key Laboratory of Reproductive Medicine, Institute of Toxicology, School of Public Health, Nanjing Medical University, Nanjing, China; ^2^ Key Laboratory of Modern Toxicology of Ministry of Education, School of Public Health, Nanjing Medical University, Nanjing, China; ^3^ Department of Cognitive Science, Yale University, New Haven, CT, United States; ^4^ Healthcare Management Program, School of Business, Nanjing University, Nanjing, China; ^5^ Department of Obstetrics, Women’s Hospital of Nanjing Medical University, Nanjing Maternity and Child Health Care Hospital, Nanjing, China

**Keywords:** endocrine disrupting chemicals, triclosan, pregnancy, thyroid, environment

## Abstract

**Objective:**

To quantitatively evaluate associations between exposure to triclosan during pregnancy and maternal thyroid hormone levels.

**Method:**

The databases of PubMed, Embase, Web of Science and Cochrane Library were systematically searched to identify relevant studies on the relationship between prenatal exposure to triclosan and maternal levels of serum thyroid hormone published before October 22, 2019. Stata 12.0 was used to examine the heterogeneity among the eligible studies.

**Results:**

Seven studies involving a total of 4,136 participants were included. Overall, descriptive analysis provided no indication that exposure to TCS during pregnancy was related to either maternal FT4 levels (ES = 0.01, 95% CI: −0.03 to 0.05, *P* = 0.00) or TSH levels (ES = −0.03, 95% CI: −0.13 to 0.07, *P* = 0.412). Although the results were statistically insignificant, with the increase of urine TCS concentration, maternal FT4 levels exhibited a tendency to increase while TSH levels had a tendency to decrease during pregnancy.

**Conclusion:**

The results indicated that exposure to triclosan during pregnancy has no significant influence on maternal levels of thyroid hormone. On account of the inconsistency of existing research designs and study locations, further studies and replication are necessary to confirm these findings.

## Introduction

Triclosan (TCS) is one of synthetic antibacterial chemical, which used widely in daily toiletries. About 96% of TCS is disposed of down residential drains (1). Although most of the TCS can be treated effectively, a small part can still be discharged to aquatic ecosystems (2). Therefore, TCS has proliferated widely in the ecological environment, eventually reaching humans through contaminated food or water (3–5). Since TCS might be absorbed by skin and oral mucosa of human easily, it can be found in various human tissues and fluids (6). It is mainly excreted in the urine, and some researches show that almost all urine samples contain TCS (7–9).

As a common environmental endocrine disrupting chemical (EDC), TCS might affect immune responses, ROS production, and cardiovascular functions (6). What is more, it may have adverse effects on the endocrine system of humans and animals, including the thyroid, one of the most important endocrine organs. The molecular structure of TCS was found to be similar to thyroid hormone (10). Thyroid hormone plays an essential role in the growth, metabolism and development of human body. Thyroid dysfunction during pregnancy may have negative effects on both the pregnant woman and fetus. The fetal thyroid gland does not begin to synthesize until the second trimester, and the adverse consequences of severe maternal TH deficiency on offspring neurodevelopment have been established. Recent evidence suggests that even more moderate forms of maternal thyroid dysfunction, particularly during early gestation, may have a long-lasting influence on child cognitive development and risk of neurodevelopmental disorders (11). In 2005, the American Thyroid Association (ATA) issued a statement on the identification, treatment and prognosis of thyroid dysfunction during pregnancy, emphasizing that further attention should be paid to possible adverse effects of thyroid dysfunction, such as clinical hypothyroidism, subclinical hypothyroidism, clinical hyperthyroidism, subclinical hyperthyroidism and thyroid autoimmune abnormalities, on pregnant women and fetuses (12–14). Many studies have indicated that maternal hypothyroidism is related to adverse pregnancy outcomes such as pre-eclampsia, spontaneous abortion, premature delivery and gestational hypertension (15–18). Also, maternal thyroid deficiency may do harm to the nerve development of offspring (19). Thyroid hormones are essential for the growth of fetus, however, hormone supply during early fetal life depends exclusively on placental to transfer various hormone from mother to fetus (20). During the first trimester, maternal thyroid hormone deficiency will have an irreversible effect on fetal growth and development of the nervous system, potentially leading to dysplasia, mental deficiency and cognitive impairment (21, 22). Moreover, according to Li et al.’s study, they concluded that intellectual development of children at 25 to 30 months is relevant to the abnormalities of maternal thyroid at 16 to 20 weeks during pregnancy (23). A few studies examined the association between exposure to TCS during pregnancy and maternal levels of thyroid hormone. In animal studies, data indicated that TCS may lead to a decrease of maternal FT3 and FT4 but have no significant association with TSH (24–28). However, among human studies, the findings were still controversial. Aker et al. suggested that prenatal exposure to TCS was associated with an increase of maternal FT4 and TSH (29). Wang et al. mentioned that there was a positive association between TCS exposure during pregnancy and maternal FT4 level (30). Some other studies found no relationship between TCS and thyroid hormone levels in pregnant women (31–33).

In our research, we systematically evaluated the relationship between prenatal exposure to TCS and maternal FT4 as well as TSH, in order to elucidate the association between TCS and maternal thyroid hormone levels and provide epidemiological evidence for reducing the incidence of thyroid disease during pregnancy.

## Materials and Methods

### Search Strategy

In our study, relevant articles were retrieved from PubMed, Embase, Web of Science and Cochrane Library on October 22, 2019 using the following search terms:

#1: (maternal) or (prenatal) or (pregnancy) or (pregnant women)

#2: (triclosan) or (TCS) or (endocrine disrupting chemicals) or (endocrine disrupter)

#3: (thyroid hormone levels) or (FT4) or (TSH) or (thyroid dysfunction) or (hypothyroidism) or (hyperthyroidism)

#4: #1 AND #2 AND #3

Furthermore, all references of relevant studies were tracked back manually to avoid oversights. Therefore, relevant research information is comprehensive.

### Inclusion and Exclusion Criteria

The inclusion criteria for the studies were:

1. Epidemiological studies based on human observation were selected, including descriptive studies, case-control studies, cohort studies and cross-sectional studies.
2. The research objects were pregnant women;
3. Prenatal exposure factor was triclosan;
4. Changes in the level of serum FT4 and TSH were measured as outcomes for study;
5. Data on the association between the risk of TCS exposure and the corresponding 95% CI was provided in the primitive study.

The exclusion criteria for the studies were:

1. Not conform to the research topic;
2. Animal studies, conference abstract, lecture literature, editorial materials or comments and so on;
3. Studies had design defects and poor quality;
4. The level of maternal thyroid hormones besides FT4 or TSH was measured;
5. Raw data was unavailable;

### Study Selection and Data Extraction

In light of the inclusion and exclusion criteria, we selected eligible articles by reviewing their titles and abstracts. In addition, the full text was reviewed for further confirmation. Relevant characteristics were extracted from selected original studies including the first author’s name, published year, research design, sample size, research time, area, correction method of TCS concentrations, outcome and 95% CI. Excel was used to record the data.

### Quality Assessment

Quality assessment of the eligible literature was made by referring to the Newcastle Ottawa Scale (NOS). An article is judged by NOS on three broad perspectives including the selection of the study subjects, comparability, exposure assessment and outcome (34). There are eight identifying items for choice, and NOS identifies high quality one with a star. A maximum of one star can be given for the “Selection” and “Exposure/Outcome” categories while a maximum of two stars can be given for each choice in the “Comparability” category.

### Statistical Analysis

Data analysis was performed using the software Stata 12.0. We extracted relevant data from eligible articles and recorded them in Excel. Statistical analysis was conducted using the meta-analysis module in Stata 12.0. The adjusted β value presented in the relevant studies was taken as the effect value, which was shown as ES (effect size).

Specific steps were as follows: (1) Heterogeneity test: Heterogeneity test was performed and it indicated a great heterogeneity among literatures. Random-effect model was used to calculate the combined effect value (35). Forest map showed effect size of each study. (2) Subgroup analysis: Subgroup analysis was performed to reduce the significant heterogeneity. (3) Sensitivity analysis: Sensitivity analysis was performed by excluding each individual article one by one to appraise the credibility of studies included in our meta-analysis.

## Result

### Literature Retrieval and Characteristics Overview

**Figure 1:**
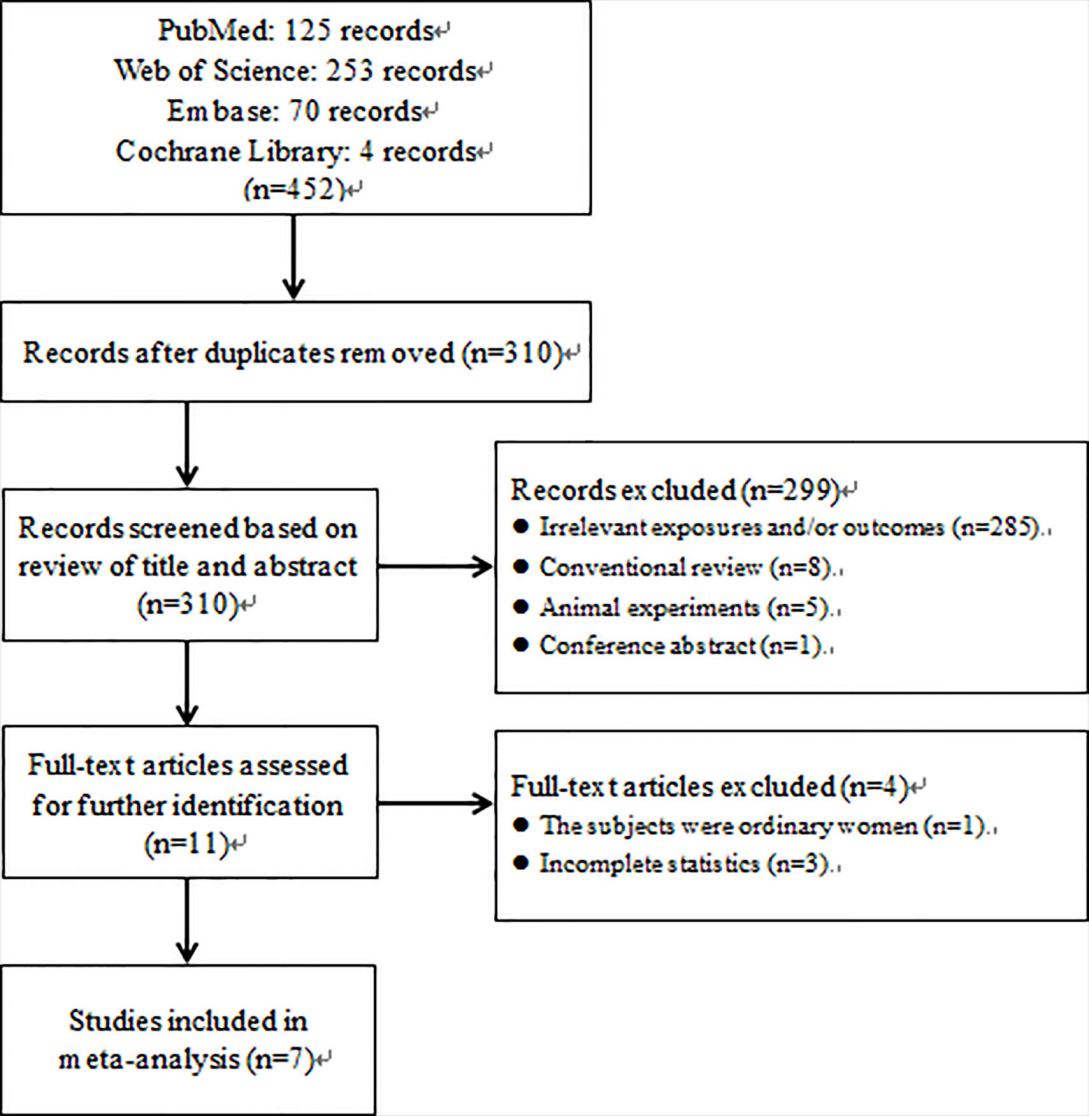
Flow diagram of selection of eligible studies.

At first, 452 studies were included by searching PubMed, Embase, Web of Science and Cochrane Library. After duplicate checking, 310 records were received. Titles and abstracts were reviewed, and 285 records were excluded on account of uncorrelated exposure factors or outcomes. 14 further records were excluded because they were not epidemiological studies. Ultimately, seven articles were included in our meta-analysis after reviewing of full-text articles (29–31, 33, 36–38)(**Figure 1**). Due to the units of FT4, TSH and exposure to TCS in the original data were not uniformed, we performed conversion of measurement units. In the articles published by Aker et al. in 2016 and 2018, we could not acquire the interquartile range (IQR) or Q_1_ and Q_3_, so we did not make a complete transformation.

**Figure 2:**
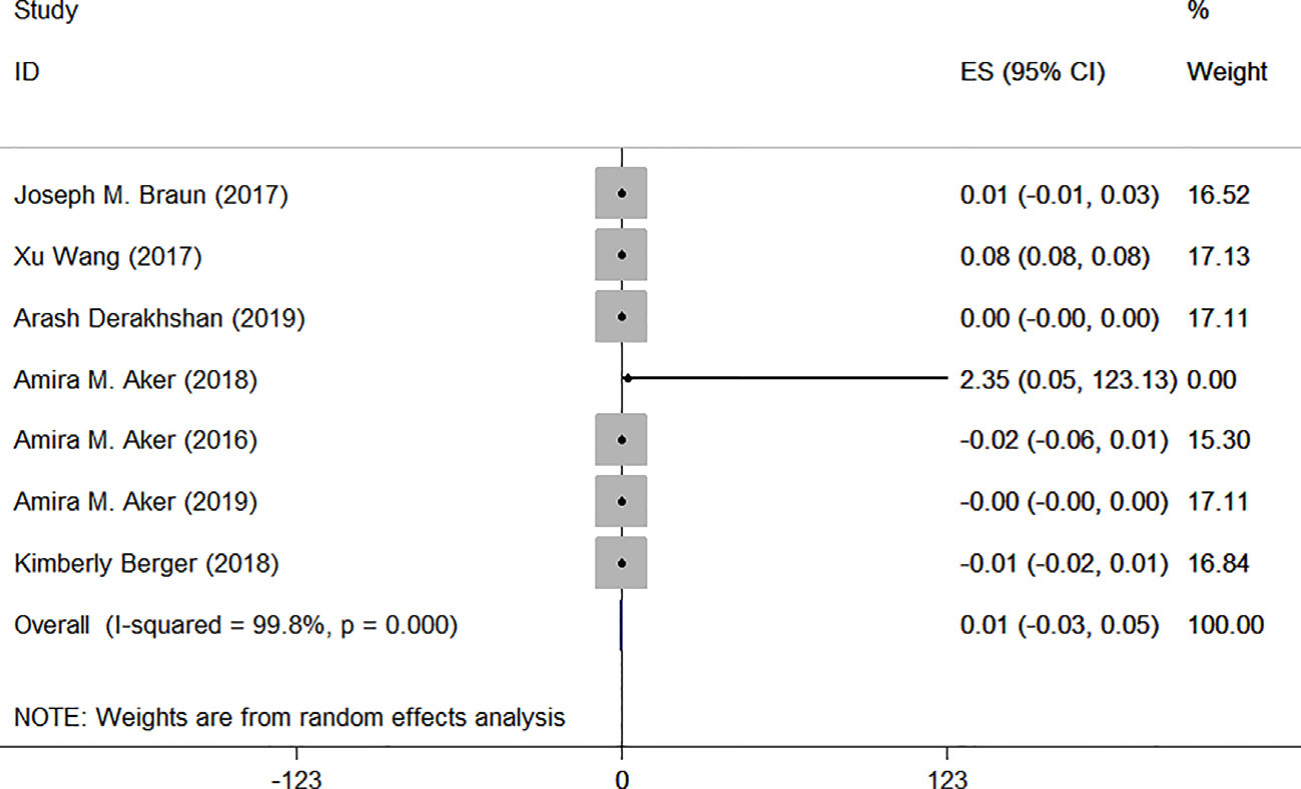
Forest plot of prenatal exposure to TCS and maternal FT4 levels.

**Figure 3:**
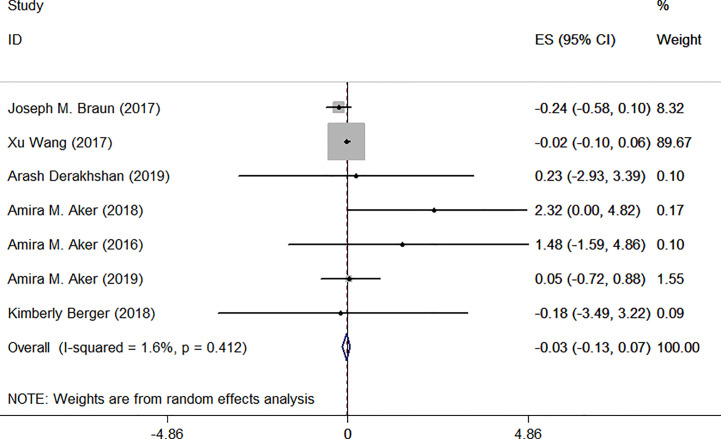
Forest plot of prenatal exposure to TCS and maternal TSH levels.

As **Table 1** shows, characteristics of the relevant studies are summarized. Only one of the studies was a nested case-control study, while the others were cohort studies. Most of the studies were located in the USA. The sample sizes ranged from 181 to 1996 people. The longest duration of the research was five years. To reduce the significant heterogeneity, subgroup analysis was performed in our meta-analysis (**Figures 2**, **3**). Articles were divided into two groups characterized by the methods used to correct urinary TCS concentrations. Three studies used urinary creatinine to standardize the urinary dilution, which accounted for differences in TCS concentrations (30, 31, 33). In the remaining studies, specific gravity was used to correct urinary biomarker concentrations (29, 36–38). We looked into the relationship between exposure to TCS during pregnancy and the level of serum TSH or FT4. We evaluated the studies by referring to the NOS. The scores were between 6 and 8.

**Table 1:** Characteristics of the articles included in our meta-analysis.

The first author’s name	Published year	Research design	Timings of thyroid hormone measurement	Timings of TCS measurement	Sample size	Research time	Area	Correction method of TCS concentrations	TSH outcome and 95%CI	FT4 outcome and 95%CI	Quality assessment
											score
Kimberly Berger (38)	2018	cohort	26.9 weeks	14.1 and 26.9 weeks	338	October 1999 to October 2000	California, USA	Specific gravity	−0.6 (−11.65, 10.75)	−0.02 (−0.065, 0.025)	6
Amira M. Aker (29)	2018	nested case-control study	9.64 weeks	9.64 weeks	439	2006 to 2008	Boston, MA,USA	Specific gravity	7.72 (0.01, 16.02)	1.22 (−2.59, 5.18)	7
			17.9 weeks	17.9 weeks							
			26.0 weeks	26.0 weeks							
			35.1 weeks	35.1 weeks							
Joseph M. Braun	2018	cohort	16 weeks	16 and 26 weeks	202	March 2003 to January 2006	Cincinnati, OH,USA	Creatinine–corrected	−0.24 (−0.58, 0.10)	0.01 (−0.01, 0.03)	7
Amira M. Aker (36)	2019	cohort	16–20 weeks and 24–28 weeks	16–20 weeks, 20–24 weeks and 24–28 weeks	602	2012 to 2017	Northern Puerto Rico, USA	Specific gravity	0.57 (−7.74, 9.63)	−0.74 (−2.45, 0.96)	8
Arash Derakhshan (33)	2019	cohort	10 weeks	10 weeks	1996	September 2007 to March 2010	Swedish	Creatinine–corrected	0.001 (−0.01272, 0.01472)	0.0 (−0.0196, 0.0196)	8
Xu Wang (30)	2017	cohort	≥28 weeks	38.8 weeks	378	June 2012 to February 2013	Shanghai, China	Creatinine–corrected	−0.02 (−0.10, 0.06)	−0.02 (−0.04, −0.001)	7
Amira M. Aker (37)	2016	cohort	16–20 weeks and 24–28 weeks	16–20 weeks, 20–24 weeks and 24–28 weeks	181	2010 to 2012	Northern Puerto Rico, USA	Specific gravity	4.90 (−5.28, 16.17)	−3.44 (−8.60, 1.71)	6

### Associations Between Prenatal Exposure to TCS and Maternal FT4 Levels

In total, seven articles were included in this meta-analysis to evaluate the effect of exposure to TCS during pregnancy on maternal FT4 levels.

**Figure 4:**
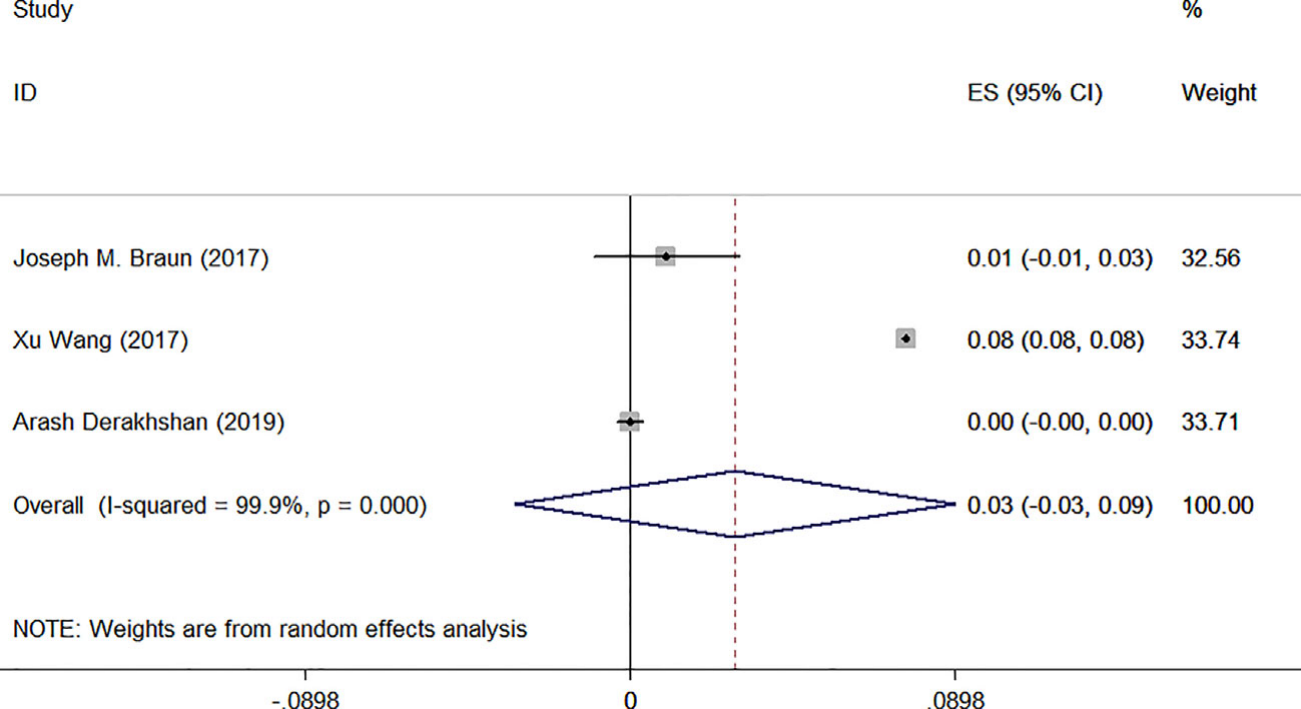
Forest plot of prenatal exposure to TCS and maternal FT4 levels standardized using urinary creatinine.

Among them, urinary creatinine was used in three studies for correction. Only one article indicated that exposure to TCS during pregnancy may lead to the increase of maternal FT4 (30), while other studies found no relationship between them. Specific results were as follows: I^2^ = 99.9% (degrees of freedom, df), *P* = 0.000. As **Figure 4** shows, ES = 0.03 (95% CI: −0.03 to 0.09), the difference was not statistically significant, which indicated that there was no association between TCS and FT4.

**Figure 5:**
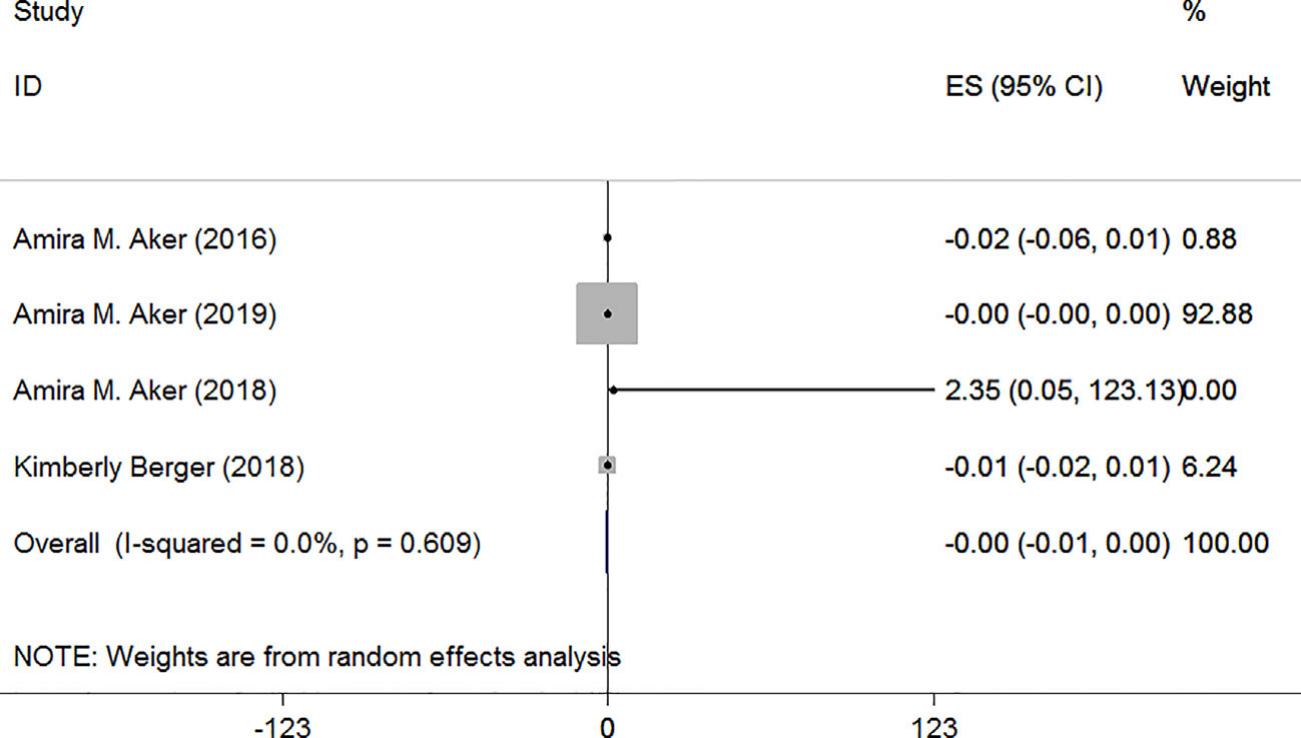
Forest plot of prenatal exposure to TCS and maternal FT4 levels standardized using specific gravity.

Specific gravity was used to correct urinary biomarker concentrations in Aker et al.’s studies and Berger et al.’s study. In the article published by Aker et al. in 2018, they suggested that maternal TCS exposure was associated with an increase of maternal FT4 (38), however, the other three studies found no association between them. Random-effect model was used due to remaining heterogeneity among these studies. The results were all negative as **Figure 5** shows: I^2^ = 0.0%, *P* = 0.609, ES = −0.00 (95% CI: −0.01 to 0.00), *P*>0.05.

### Associations Between Prenatal Exposure to TCS and Maternal TSH Levels

The same seven articles were combined to determine whether prenatal exposure to TCS is associated with maternal TSH levels. Similarly, studies were divided into two groups according to the method of correction.

**Figure 6:**
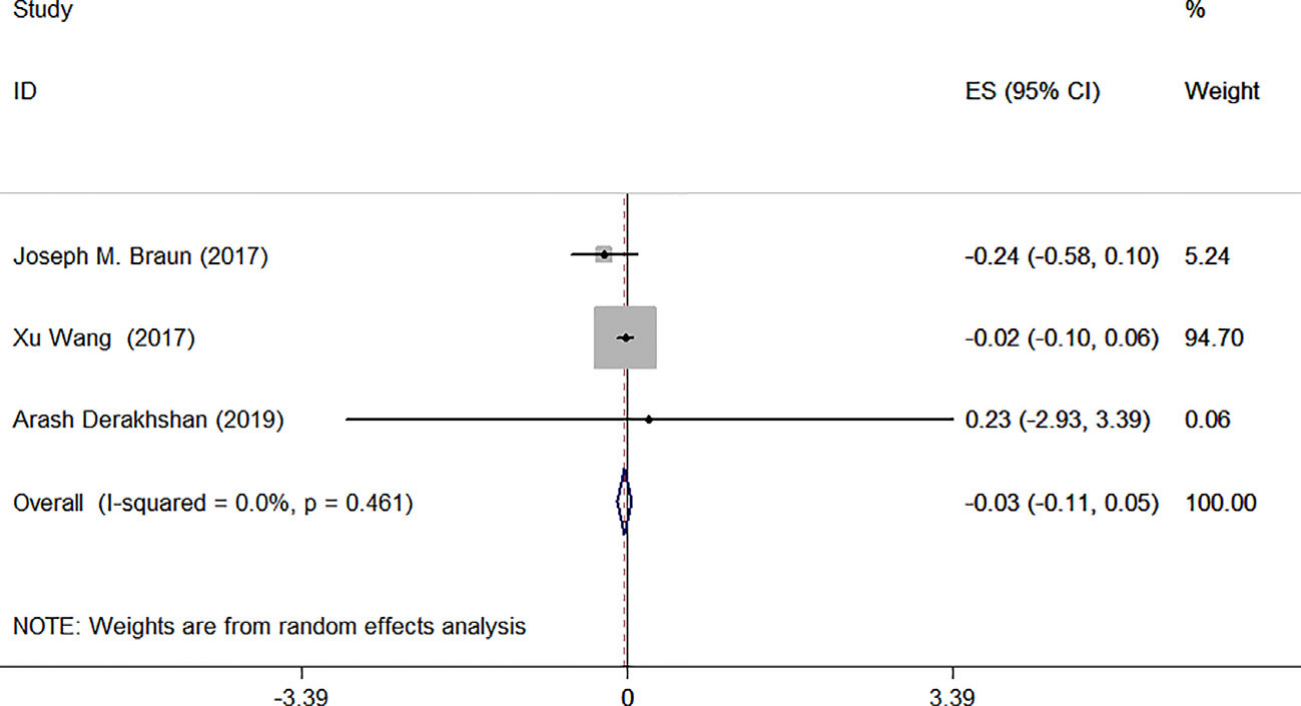
Forest plot of prenatal exposure to TCS and maternal TSH levels standardized using urinary creatinine.

Urinary creatinine was used to standardize for urinary dilution in three studies. We used random-effect model due to remaining heterogeneity among these three studies. Meta-analysis forest plot was shown in **Figure 6**: I^2^ = 0.0%, *P* = 0.461, ES = −0.03 (95% CI: −0.11 to 0.05), *P*>0.05. The difference was not statistically significant.

**Figure 7:**
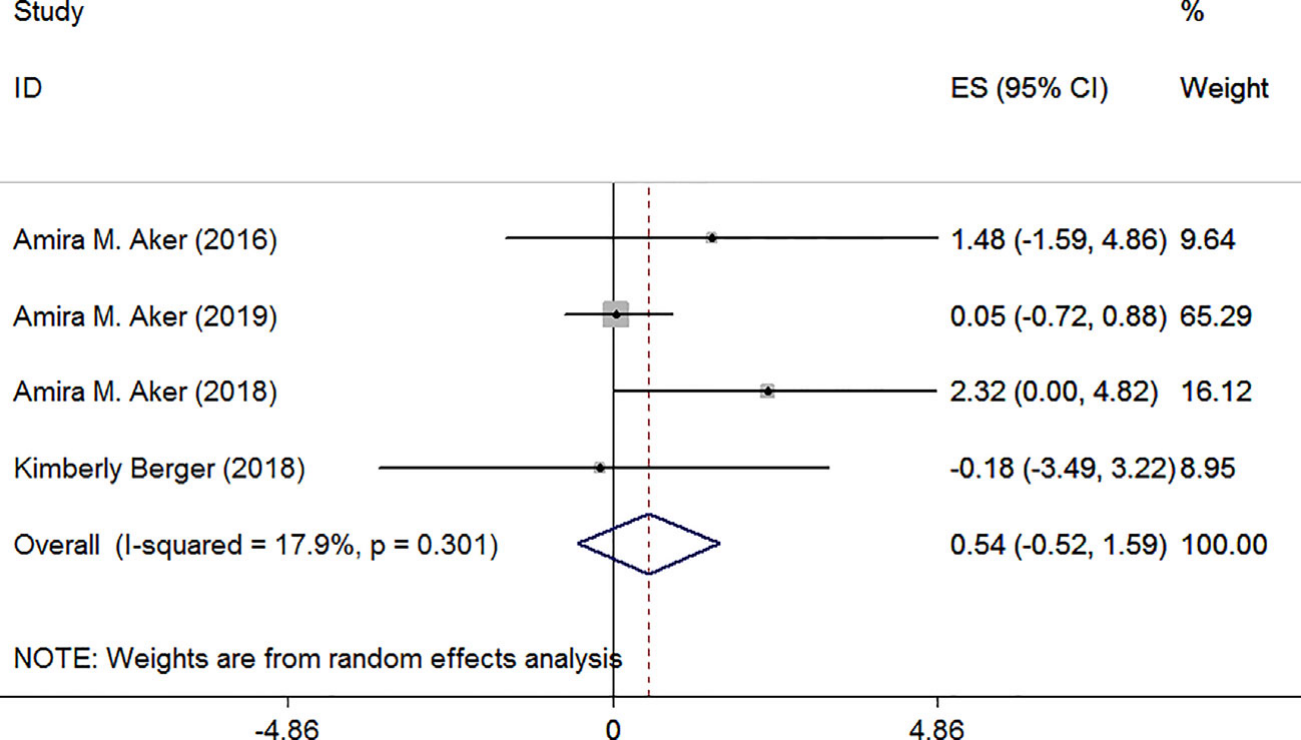
Forest plot of prenatal exposure to TCS and maternal TSH levels standardized using specific gravity.

The remaining articles were corrected using specific gravity. Only one article identified a positive relationship between TCS and TSH (29). Meta-analysis forest plot was shown in **Figure 7**: I^2^ = 17.9%, *P* = 0.301, ES = 0.54 (95% CI: −0.52 to 1.59), *P*>0.05. The result indicated that there was no association between TCS and TSH.

### Sensitivity Analysis

**Figure 8:**
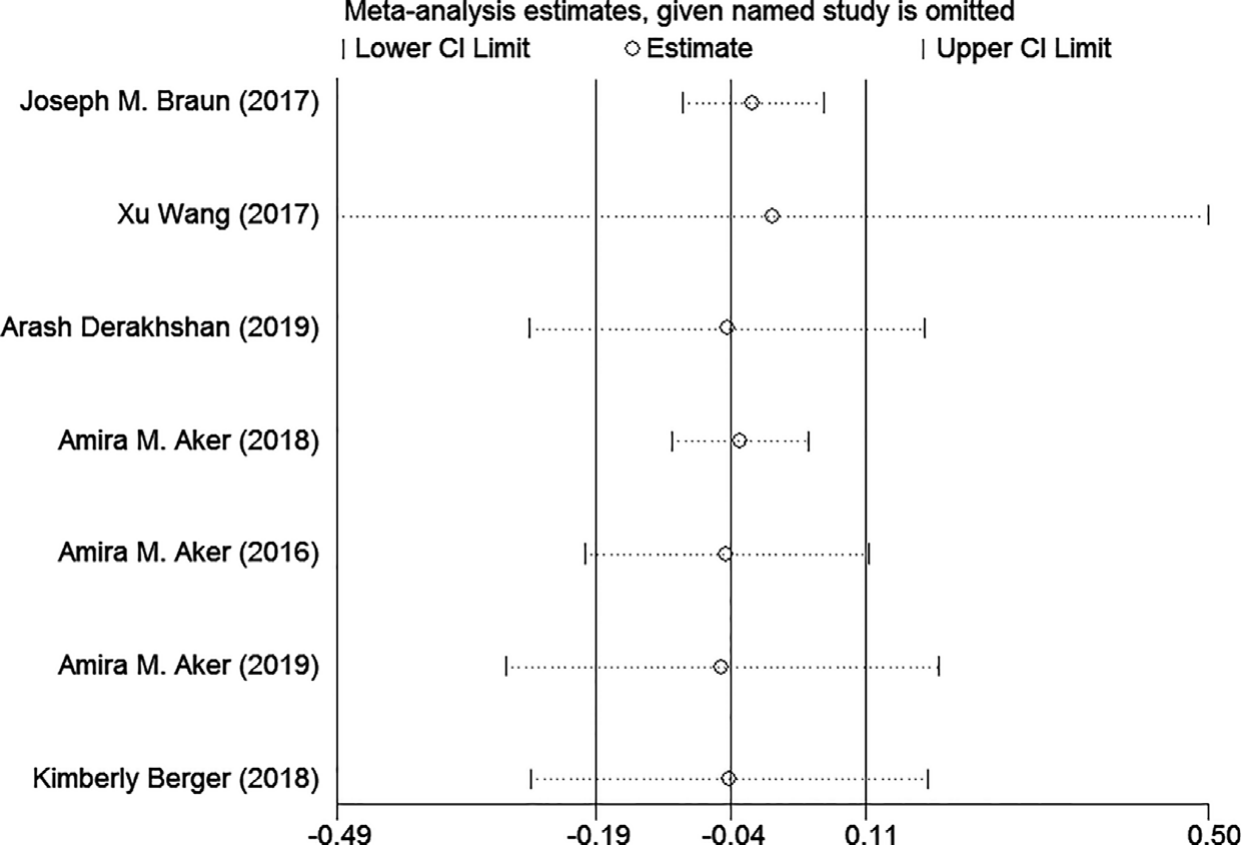
Sensitivity analysis of prenatal exposure to TCS and maternal thyroid hormone levels.

In our study, sensitivity analysis was performed by excluding each individual article one by one. The results did not show evident differences when we removed any other studies, which suggested the credibility of studies included in our meta-analysis (**Figure 8**).

## Discussion

Our study is the first meta-analysis reviewing the current evidence to investigate whether exposure to TCS during pregnancy can affect maternal thyroid hormone levels. This meta-analysis included seven studies and 4,136 participants. As the performed sensitivity analysis shown, the results were fairly consistent before and after we excluded articles one by one. In our meta-analysis, maternal FT4 levels exhibited a tendency to increase while levels of TSH had a tendency to decrease with higher urine TCS concentration. As evidence shows in Andersen et al.’s study, abnormal maternal thyroid function in early pregnancy was associated with epilepsy, ASD, and ADHD in the child (39), which we should pay more attention to.

We performed subgroup analysis to reduce the significant heterogeneity in our study, which can mainly be attributed to differences in calibration methods of urinary TCS concentrations, control of potential confounders, and timing of TCS concentration and thyroid hormone measurements. Above all, the correction methods of TCS concentrations in maternal urine were different among the included articles. Urinary creatinine was used in three studies to standardize for urinary dilution (30, 31, 33). In Aker et al.’s studies and Berger et al.’s study, specific gravity was used to correct urinary biomarker concentrations (29, 36–38). Therefore, we encourage that further research should be undertaken to determine which method is more precise. In addition, potential confounders such as area, maternal age and BMI were not the same in different articles. Five studies were performed in the USA (29, 31, 36–38), one study was conducted in China (30), and another one was carried out in Sweden (33). In spite of the statistical data being adjusted, there might still be some information bias. Important covariates were ignored in some studies, such as household income, marital status and maternal country of birth. These may influence the correlation between TCS exposure during pregnancy and maternal levels of serum thyroid hormone. Ultimately, timing of TCS concentration and thyroid hormone measurements may also lead to heterogeneity. We found that timing of TCS concentration and thyroid hormone measurements were different among the articles. In Aker et al.’s studies, TCS concentrations and thyroid hormone levels were measured twice during the second trimester (36, 37), while in the other study, they were measured four times during the whole pregnancy (29). Wang et al. measured TCS concentrations and thyroid hormone levels in the third trimester (30). In the article published by Braun et al. in 2017 and another one published by Berger et al. in 2018, the measuring times were both in the second trimester (31, 38). However, Derakhshan et al. only measured during the first trimester (33). Therefore, we suggest that further studies investigate the impact of the timing of TCS and thyroid hormone measurements on maternal thyroid hormone levels.

Significant associations were shown in two articles. In Wang et al.’s study, they suggested a significant association between maternal exposure to TCS and prenatal FT4. It is the only article measuring TCS concentration in urine during the third trimester. Therefore, we hypothesize that pregnant women are more sensitive to TCS in the third trimester, and more measurements during different trimesters should be taken. In addition, this study is the only one which was performed in Asia. As a result, we suggested that different races may have different sensitivity levels to TCS, and more studies on identifying populations sensitive to TCS should be performed by simulating toxicokinetic variability (40). The article published by Aker et al. in 2018 found a positive correlation between exposure to TCS during pregnancy and TSH and FT4 levels. Due to the interquartile range (IQR) or Q1 and Q3 could not be acquired in this article, completed transformation was unable to be performed. Additional studies are required for further confirmation.

In animal tests, exposure to TCS during pregnancy resulted in significant FT4 reduction but TSH levels were not obviously changed (25–27, 41). As Paul et al. reported in 2010, oral exposure of TCS (0, 30, 100 and 300 mg/kg/day) was performed on female rats from the sixth day of pregnancy to 21 days postpartum, and the level of TT4 was detected in female mice at 22 days postpartum. TT4 levels of the female rats in the 300 mg/kg/day exposure group decreased by about 30%, and no significant change was found in other exposure groups (27). In population studies, the results were statistically insignificant. Considering that the concentration of TCS in nature was far below that of animal studies, we accounted that the significant reduction of FT4 was related to the exposure concentration.

As the results illustrated, exposure to TCS during pregnancy was not associated with either maternal FT4 or maternal TSH. However, considering the diversity of methods used in different studies and limitations in the quantity of current research, the results should be treated with caution. Therefore, experimental design methods should be optimized, and further studies are needed to investigate the relationship between exposure to TCS during pregnancy and maternal thyroid hormone levels.

## Conclusion

In our meta-analysis, we concluded that there was no association between exposure to TCS during pregnancy and maternal thyroid hormone levels. Most of the included articles showed insignificant connections, but there were still a few articles that indicated that exposure to TCS during pregnancy may lead to the increase of maternal FT4 and TSH. Consequently, we recommend that larger-scale and more extensive cohort studies should be done to determine the relationship between prenatal exposure to TCS and maternal thyroid hormone levels.

## Data Availability Statement

The raw data supporting the conclusions of this article will be made available by the authors, without undue reservation.

## Author Contributions

WL and ZS conceived the study. DC collected and organized the data. JL and WY drafted the manuscript. KF and YX helped refine the manuscript. All authors contributed to the article and approved the submitted version.

## Funding

This work was supported by the National Natural Science Foundation of China (81571458, 81100436) and Six talent peaks project in Jiangsu Province (WSW-121).

## Conflict of Interest

The authors declare that the research was conducted in the absence of any commercial or financial relationships that could be construed as a potential conflict of interest.
